# Prognostic Value of Immunoscore and PD-L1 Expression in Metastatic Colorectal Cancer Patients with Different RAS Status after Palliative Operation

**DOI:** 10.1155/2018/5920608

**Published:** 2018-01-31

**Authors:** Ruiqi Liu, Ke Peng, Yiyi Yu, Li Liang, Xiaojing Xu, Wei Li, Shan Yu, Tianshu Liu

**Affiliations:** Department of Oncology, Zhongshan Hospital, Fudan University, Shanghai 200032, China

## Abstract

Colorectal cancer (CRC) is the fifth leading cause of cancer death and the fifth most commonly diagnosed cancer in China. Approximately, 25% of CRC was in the advanced stage as diagnosed, and 40% of patients with CRC progress to metastatic colorectal cancer (mCRC). RAS mutation status is now routinely used to select their therapy. But it is still a question whether RAS mutation status is a prognostic marker. In our study, we detected RAS mutation, immunoscore (IS), and PD-L1 expression in 60 Chinese mCRC patients who received palliative operation. The Kaplan-Meier survival analysis showed that the overall survival (OS) in patients with RAS wild type was better than those with RAS mutated type. Moreover, in multivariate analysis, RAS mutation and PD-L1 expression were demonstrated to be the independent negative prognostic factors for OS (*P* = 0.044, HR: 0.258, and 95% CI: 0.069–0.967; *P* = 0.048, HR: 0.276, and 95% CI: 0.077–0,988). All results suggested that, combined with IS, PD-L1 expression and RAS status may be the prognostic indicators for mCRC patients with palliative operation.

## 1. Introduction

The World Health Organization (WHO) showed nearly half of colorectal cancer (CRC) cases are detected in Asia, mostly in China. CRC was the fifth most commonly diagnosed cancer in China [1], with more than 0.3 million new cases and 191000 deaths occurring [2]. In the last few years, the mortality of CRC was declining in United States but rapidly growing in China, which is the fifth leading cause of cancer death. Furthermore, approximately 25% of CRC was in the advanced stage as diagnosed and more than 40% of patients with CRC progress to metastatic colorectal cancer (mCRC) [3].

The RAS protooncogenes encode a family of highly homologous proteins, including HRAS, KRAS, and NRAS. They are involved in RAS/RAF/MEK/ERK signal pathway, which regulates the growth and survival properties of cells [4]. For mCRC patients, RAS mutation is usually used as an important predictive factor for the clinical response of anti-EGFR treatment. Recent studies have demonstrated that BRAF mutations are related to poor prognosis of mCRC [5–7]. However, we could not draw a firm conclusion about the correlation between the RAS mutation and the prognosis in mCRC patients with palliative operation.

Tumor-infiltrating immune cells, which play a role in recognition and elimination of tumor cell, have been reported to promote immune evasion and metastasis in CRC [8, 9]. Recently, several studies have demonstrated that immunoscore (IS), based on the density of CD8+ and CD3+ tumor-infiltrating lymphocytes in the invasive margin and the core of tumor, is vastly thought to be superior to the current tumor-node-metastases (TNM) staging system [10, 11]. However, the evidence is limited for mCRC.

Programmed cell death-ligand 1 (PD-L1) has been reported to function in the immunoregulatory system during certain conditions, including autoimmune disease, allograft rejection, pregnancy, and cancer [12]. Several studies suggested that PD-L1 expression in lymphocyte cells and in tumor cells of CRC is related to a high density of tumor-infiltrating immune cells [13, 14]. Hence, expression levels of PD-L1 were inversely correlated to T-cell densities in CRC tissue. However, the complex interrelationship between prognostic of mCRC and PD-L1 expression is still unknown.

Although most studies have demonstrated that BRAF mutations are related to poor prognosis of mCRC, we could not draw a firm conclusion about the correlation between the RAS mutation and the prognosis in mCRC patients. The objectives of this study were to confirm the prognostic value of the immunoscore of CD3+CD8 and the PD-L1 expression in mCRC with or without RAS mutation.

## 2. Materials and Method

### 2.1. Patients

This retrospective study included 60 mCRC patients with palliative operation at diagnosis between December 2013 and March 2016. Available variables included the following: sex, age of diagnosis, tumor location, RAS mutation type, histological type, vascular and perineural invasion, and metastatic sites. All patients were followed up until their deaths, or their last follow-up, or March 31, 2017. We defined the overall survival (OS) as the time from the date of primary treatment to the date of the last follow-up.

### 2.2. Immunohistochemistry and Image Analysis of Tumor-Infiltrating Immune Cell

The presence of tumor-infiltrating immune cells was confirmed by immunohistochemistry using antibodies for CD3 (ZA-0503), CD8 (ZA-0508), and PD-L1 (ab205921). Immunostaining for CD3 and CD8 and PD-L1 was performed using a Bond polymer kit (Leica Microsystems) and Leica BONDMAX autostainer (Leica Microsystems). All immunostained slides were scanned on an Aperio ScanScope® CS instrument (Aperio Technologies, Inc., Vista, CA, USA). The immunomarker-positive tumor-infiltrating immune cells were quantified by computerized image analysis system, ImageScope™ (Aperio Technologies). CD3+, CD8+, and PD-L1+ lymphocytes were counted using the Nuclear v9 algorithm. The density of immune infiltrates was obtained from the entire area of the tissue core.

### 2.3. Determination of Scoring System

Immunoscore (IS) was performed as described before [15]. Briefly, immunomarker-positive tumor-infiltrating immune cells were quantified by computerized image analysis system, ImageScope (Aperio Technologies). CD3+ and CD8+ lymphocytes were counted using the Nuclear v9 algorithm. We used the same cut-off values as Kwak et al. described. IS was defined as a quantification system based on the combination of two markers (CD3 and CD8) in two regions—the core of tumor (CT) and the invasive margin (IM) [14, 16]. A high density of immune marker positive lymphocytes in each region was recorded as a score. IS is a summation of the score of CD3+ and CD8+ TILs in the CT and IM, which is from 0 to 4. Then, all the patients could be divided into two groups—IS low group (0, 1, and 2) or high group (3, 4).

### 2.4. Statistics

All data were statistically analyzed by the Statistical Package for the Social Sciences, version 23.0 (SPSS Inc., Chicago, IL, USA). The correlation among clinicopathological features and mutation was calculated by a Chi-square test (for categorical variables) and Student *t*-test (for continuous variables). Overall survival was calculated by the Kaplan-Meier method. For identifying the independent prognostic factors for OS, the Cox proportional-hazards model was used for univariate and multivariate analyses. *P* value less than 0.05 was considered to be statistically significant.

## 3. Results

### 3.1. Basic Characteristics of the Recruited mCRC Patients

We analyzed the basic characteristics of the recruited mCRC patients (Table 1). We found RAS gene mutant tumors were more likely to develop in the right colon in comparison with RAS wild-type tumors (68.75% versus 31.09%, *P* = 0.017). PD-L1 was more likely to express in the rectum in comparison with colon (68.00 versus 25.71%, *P* = 0.001).

### 3.2. Survival Analysis Associated with RAS Status

**Figure 1:**
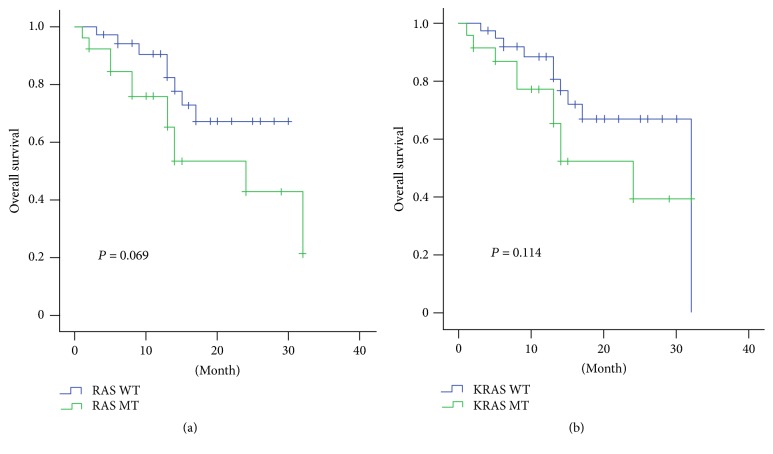
*Relationship of RAS status and overall survival in mCRC*. (a) Overall survival analysis to RAS statue of all the patients. (b) Overall survival analysis to KRAS statue of all the patients.

We sequenced all coding exons of all three RAS isoforms in the 60 mCRCs at first. The Kaplan-Meier survival analysis demonstrated that there were no significant differences in OS between RAS (*P* = 0.069), KRAS (*P* = 0.114), mutation type and wild type (Figures 1(a) and 1(b)).

### 3.3. Prognostic Value of Immunoscore in mCRCs

**Figure 2:**
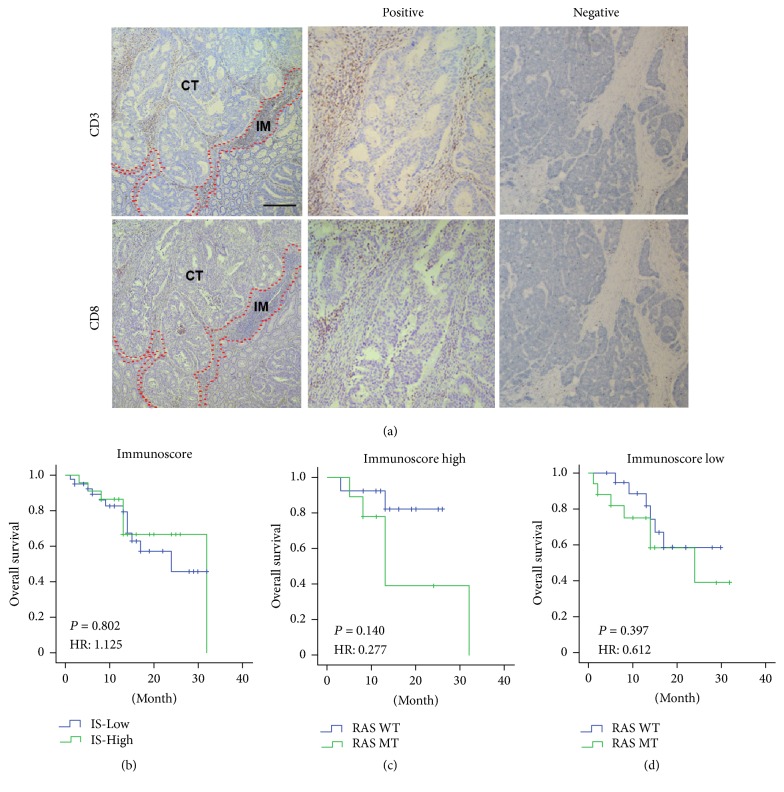
*Prognostic value of immunoscore in mCRCs.* (a) The immunohistochemical results of the CD3 and CD8 in the CT and IM of the primary tumor. (b) The Kaplan-Meier survival curve according to IS. (c) The Kaplan-Meier survival curve according to RAS statue in IS-High patients. (d) The Kaplan-Meier survival curve according to RAS statue in IS low patients.

The immunohistochemical results of the CD3 and CD8 were showed in Figure 2(a). IS is a summation of the score of CD3+ and CD8+ TILs in the CT and IM, which is from 0 to 4. Then, all the patients were divided into two groups—IS low group (0, 1, and 2) and high group (3, 4). The Kaplan-Meier analysis showed immunoscore (IS) was not significantly correlated with survival (*P* = 0.799) (Figure 2(b)).

Then, we divided these patients into two groups by IS. The Kaplan-Meier analysis shows RAS gene type was not significantly correlated with survival in each group (*P* = 0.101, *P* = 0.387, resp.). But, by univariate COX regression analysis, the *P* value and hazard ratios were 0.140 and 0.277 in IS-High group (Figures 2(c) and 2(d)).

### 3.4. Prognostic Value of PD-L1 Expression in mCRCs

**Figure 3:**
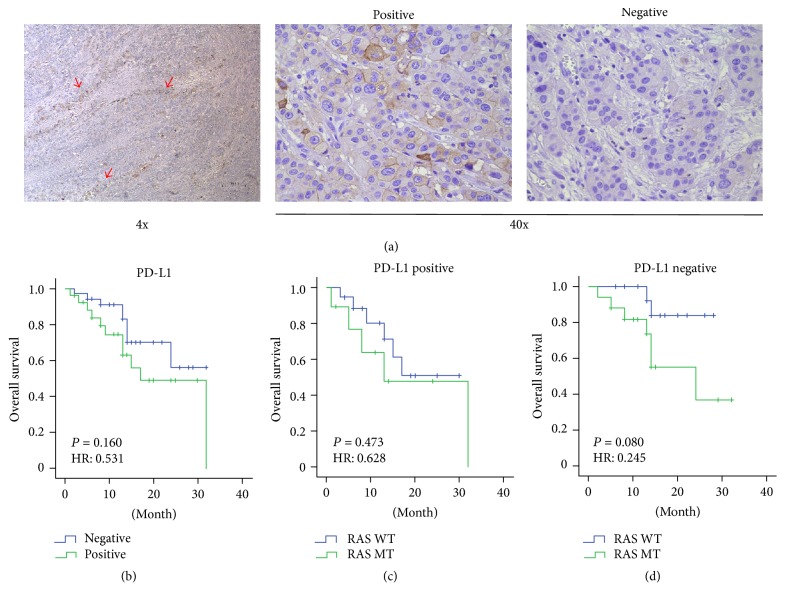
*Prognostic value of PD-L1 expression in mCRCs. *(a) The PD-L1 expression of the primary tumor. (b) The Kaplan-Meier survival curve according to PD-L1 expression. (c) The Kaplan-Meier survival curve according to RAS statue in patients with PD-L1 expression. (d) The Kaplan-Meier survival curve according to RAS statue in patients without PD-L1 expression.

The immunohistochemical results of the PD-L1 expression were showed in Figure 3(a). All the patients were divided into two groups with or without the expression of PD-L1. The Kaplan-Meier analysis showed the PD-L1 expression was not significantly correlated with survival (*P* = 0.143) (Figure 3(b)).

Then, we divided these 60 patients into another two groups by PD-L1 expression. The Kaplan-Meier analysis showed RAS gene type was not significantly correlated with survival in each group, either (*P* = 0.287, *P* = 0.052, resp.). But, by univariate COX regression analysis, the *P* value and hazard ratios were 0.080 and 0.24 in PD-L1-negative group (Figures 3(c) and 3(d)).

### 3.5. Univariate and Multivariable Analyses in mCRCs

We used the Cox proportional-hazards model to investigate the independent prognostic factors for OS in patients with mCRC (Table 2). The univariate analysis showed that the OS of patients with RAS mutation was worse than patients without RAS mutation (hazard ratio (HR): 0.473), though the *P* value is not significant (*P* = 0.069). In multivariate analysis, RAS mutation and PD-L1 expression in lymphocyte were demonstrated to be the independent negative prognostic factor for OS (*P* = 0.044, HR: 0.258, and 95% CI: 0.069–0.967; *P* = 0.048, HR: 0.276, and 95% CI: 0.077–0,988). And both IS and age had impressive influence on OS (HR: 2.681; HR: 2.127).

## 4. Discussion

In this study, we elucidated the prevalence of RAS mutations in Chinese mCRC patients, clarified the correlation between clinicopathological features and gene status, and investigated the prognostic value of tumor-infiltrating cells. So far, most clinical evidence about RAS and BRAF mutations in mCRC were originated from western countries. In this paper, we detected the frequency of RAS and KRAS mutation in 60 Chinese mCRC patients with palliative operation (53.33%, 38.33%). More recently, several reports have shown that exon 3 or 4 mutation of KRAS and exons 2–4 mutation of NRAS occurred in approximately 10 percent of mCRC patients with KRAS exon 2 wild-type tumors. Our data showed that the frequency of patients with KRAS exon 2 mutant tumors is similar.

As previously reported, the presence of BRAF mutations in CRC was always a strongly poor prognostic marker for clinical outcome. And patients with BRAF mutant are often refractory to systematic chemotherapy [17]. However, there was no identical conclusion about the correlation between the RAS mutation and the prognosis in mCRC patients. Previously, research showed that there was insufficient evidence to definitively state that patients with RAS mutations mCRC could benefit from bevacizumab combined with chemotherapy as first-line treatment [18]. Recently, several studies have demonstrated that immunoscore (IS) has high prognostic utility, which could be demonstrated as the density of CD3+ and CD8+ lymphocytes in the tumor center (CT) and invasive margin (IM) [16, 19, 20]. Moreover, it has been reported that the IS method is much better while compared to the current tumor-node-metastases (TNM) staging system, especially in colon cancers [21]. In a recent report, Lea et al. described the limitations of the current TNM staging system in predicting the outcome of patients with CRC [22]. They suggested that the immune cell density in the stromal environment could be a better prognostic marker. This suggestion was also confirmed by Mlecnik et al. [23]. Furthermore, the multivariate survival analysis conducted by Anitei et al. confirmed that the IS system has stronger prognostic value than the TNM staging system [24]. In this study, all the patients were mCRC with palliative operation and we demonstrated the prognostic value of the IS method. We divided all the patients to low IS (0, 1, and 2) and high group (3, 4). Our study demonstrated that patients without RAS mutation have a better prognostic in the higher density of CD3+ and CD8+ lymphocytes group. Most of the studies have demonstrated that dense infiltration of CD3+ and CD8+ lymphocytes is associated with less aggressive clinic-pathological features and a better prognosis [24, 25]. Hence, the IS system could be a robust prognostic factor that is assessable for mCRC patients without RAS mutation.

Previous study suggested that the activation of the PD-1/PD-L1 signaling pathway created an immunosuppressive tumor microenvironment for tumors to escape from immune clearance [26]. Thus, blockade of the PD-1/PD-L1 function provided a potential strategy for cancer immunotherapy. Many clinical trials have been conducted to show the clinical benefit of various types of tumors from anti-PD-1/PD-L1 immunotherapy, such as malignant melanoma, non-small cell lung cancer, and renal cell carcinoma [27, 28]. A recent phase II trial reported that mismatch-repair status could predict a survival benefit during blockade of the immune checkpoint system in CRC patients [29]. Interestingly, several studies found that PD-L1 expression was also correlated to MSI status [30]. In our study, we found that high IS correlated with prolonged OS and was a good independent prognostic indicator in RAS wild-type mCRC patients. According to other research, high PD-1 expression has been correlated with improved response to immune checkpoint inhibitors, compared with low PD-L1 expression. Furthermore, PD-L1 expression on the peritumor cells may be correlated with improved response to immune checkpoint inhibitors. In addition, the high mutational frequency found within tumors raises the possibility that T cells may preferentially invade tumors in patients whose T cells recognize mutated epitopes found within the tumor tissue [31]. These findings suggest that PD-L1 expression is a useful and reproducible tool for predicting survival for mCRC patients. In our study, we divided the 60 mCRC patients into two groups according to the percent of PD-L1 expression in tumor cell and lymphocytes. The Kaplan-Meier analysis showed that there was a better prognostic with PD-L1 expression in wild-type RAS patients. We found that the PD-L1 expression was the independent negative prognostic factor for OS in multivariate analysis (*P* = 0.048, HR: 0.276, and 95% CI: 0.077–0.988).

## 5. Conclusions

In conclusion, for the mCRC patients with palliative operation and negative PD-L1 expression, the RAS mutation is a negative prognostic factor. And the RAS mutation maybe a potential negative prognostic factor for the mCRC patients with palliative operation and high immunoscore. All the results suggested that, combined with RAS status, IS and PD-L1 expression may be the prognostic indicators for mCRC patients with palliative operation. This will provide a better prognostic marker for the treatment of mCRC patients without radical operation.

## Figures and Tables

**Table 1:** Basic characteristics of the recruited mCRC patients.

*Characteristics*	Total	*RAS mutation*			*Immunoscore*			*PD-L1 expression*		
		Mutation type	Wild type	*P* value	Low	High	*P* value	Negative	Positive	*P* value
*Patients number (percentage)*	60	26 (43.33%)	34 (56.67%)		38 (63.33%)	22 (36.67%)		34 (56.67%)	26 (43.33%)	
*Age*				0.341			0.325			0.207
Mean ± SD		59.64 ± 10.68	59.15 ± 10.51		59.84 ± 10.16	59.22 ± 11.53		59.41 ± 8.85	59.88 ± 12.69	
*Sex*				0.832			0.294			0.429
Male	43	19	24		29	14		23	20	
Female	17	7	10		9	8		11	6	
*Location*				0.054			0.851			0.002
Right	16	11	5	0.017	11	5	0.600	10	6	0.582
Left	19	7	12		12	7		16	3	
Rectum	25	8	17	0.134	15	10	0.651	8	17	0.001
*Site of metastasis*										
Liver	53	22	31	0.433	34	19	0.718	30	23	0.978
Lung	11	7	4	0.133	7	4	0.982	8	3	0.234
Others	4	2	2	0.781	2	2	0.567	2	2	0.781

Age was compared between two groups by using independent *t*-test; *P* values are calculated by using Fisher's exact test because less than 80% of the cells have an expected frequency of 5 or greater, or any cell has an expected frequency smaller than 1.0.

**Table 2:** Univariate and multivariate analyses of OS in 60 mCRC patients.

Variables	Univariate analysis			Multivariate analysis		
	*P* value	HR	95% CI	*P* value	HR	95% CI
Age (≥60)	0.079	2.255	0.909–5.597	0.166	2.127	0.731–6.188
Location (left/right)	0.714	0.826	0.298–2.292	0.534	0.631	0.148–2.691
RAS mutation	0.109	0.473	0.189–1.181	0.044	0.258	0.069–0.967
Histology	0.228	0.551	0.209–1.453	0.467	0.643	0.195–2.114
Nerve invasion	0.587	1.293	0.512–3.265	0.954	0.969	0.334–2.812
Vascular invasion	0.719	1.203	0.440–3.285	0.613	0.734	0.221–2.433
Immunoscore	0.802	1.125	0.447–2.831	0.127	2.681	0.756–9.507
PD-L1	0.160	0.531	0.219–1.284	0.048	0.276	0.077–0.988

## Abbreviations

CRC: Colorectal cancer
CT: Core of tumor
HR: Hazard ratio
IM: Invasive margin
IS: Immunoscore
mCRC: Metastatic colorectal cancer
MT: Mutant type
OS: Overall survival
PD-L1: Programmed cell death-ligand 1
TILs: Tumor-infiltrating lymphocytes
TNM: Tumor node metastases
WT: Wild type.

## References

[1] Tao F., Lv J., Wang W., Jin K. (2015). Current management of colorectal hepatic metastasis. *International Journal of Clinical and Experimental Medicine*.

[2] Chen W., Zheng R., Baade P. D. (2016). Cancer statistics in China, 2015. *CA: A Cancer Journal for Clinicians*.

[3] Zabala M., Alzuguren P., Benavides C. (2009). Evaluation of bioluminescent imaging for noninvasive monitoring of colorectal cancer progression in the liver and its response to immunogene therapy. *Molecular Cancer*.

[4] Negru S., Papadopoulou E., Apessos A. (2014). KRAS, NRAS and BRAF mutations in Greek and Romanian patients with colorectal cancer: A cohort study. *BMJ Open*.

[5] Van Cutsem E., Peeters M., Siena S. (2007). Open-label phase III trial of panitumumab plus best supportive care compared with best supportive care alone in patients with chemotherapy-refractory metastatic colorectal cancer. *Journal of Clinical Oncology*.

[6] McCubrey J. A., Steelman L. S., Abrams S. L. (2006). Roles of the RAF/MEK/ERK and PI3K/PTEN/AKT pathways in malignant transformation and drug resistance. *Advances in Enzyme Regulation*.

[7] Scaltriti M., Baselga J. (2006). The epidermal growth factor receptor pathway: A model for targeted therapy. *Clinical Cancer Research*.

[8] Di Caro G., Marchesi F., Laghi L., Grizzi F. (2013). Immune cells: plastic players along colorectal cancer progression. *Journal of Cellular and Molecular Medicine*.

[9] Giraldo N. A., Becht E., Remark R., Damotte D., Sautès-Fridman C., Fridman W. H. (2014). The immune contexture of primary and metastatic human tumours. *Current Opinion in Immunology*.

[10] Fridman W. H., Galon J., Dieu-Nosjean M.-C. (2011). Immune infiltration in human cancer: prognostic significance and disease control. *Current Topics in Microbiology and Immunology*.

[11] Galon J., PagΦs F., Marincola F. M. (2012). Cancer classi cation using the Immunoscore: a worldwide task force. *Journal of Translational Medicine*.

[12] Gabrielson A., Wu Y., Wang H. (2016). Intratumoral CD3 and CD8 T-cell densities associated with relapse-free survival in HCC. *Cancer Immunology Research*.

[13] Mlecnik B., Bindea G., Angell H. K. (2016). Integrative Analyses of Colorectal Cancer Show Immunoscore Is a Stronger Predictor of Patient Survival Than Microsatellite Instability. *Immunity*.

[14] Böger C., Behrens H.-M., Mathiak M., Krüger S., Kalthoff H., Röcken C. (2016). PD-L1 is an independent prognostic predictor in gastric cancer of Western patients. *Oncotarget *.

[15] Kwak Y., Koh J., Woo D., et al. (2016). KimImmunoscore encompassing CD3+ and CD8+ T cell densities in distant metastasis is a robust prognostic marker for advanced colorectal cancer. *Oncotarget*.

[16] Kwak Y., Koh J., Kim D.-W., Kang S.-B., Kim W. H., Lee H. S. (2016). Immunoscore encompassing CD3+ and CD8+ T cell densities in distant metastasis is a robust prognostic marker for advanced colorectal cancer. *Oncotarget*.

[17] Galon J., Mlecnik B., Bindea G. (2014). Towards the introduction of the 'Immunoscore' in the classi cation of malignant tumours. *The Journal of Pathology*.

[18] Siena S., Rivera F., Taieb J. (2017). Survival Outcomes in Patients With RAS Wild Type Metastatic Colorectal Cancer Classified According to Köhne Prognostic Category and BRAF Mutation Status. *Clinical Colorectal Cancer*.

[19] Zhou M., Yu P., Qu J. (2016). Efficacy of Bevacizumab in the First-Line Treatment of Patients with RAS Mutations Metastatic Colorectal Cancer: A Systematic Review and Network Meta-Analysis. *Cellular Physiology and Biochemistry*.

[20] Fridman W. H., Pagès F., Sautès-Fridman C., Galon J. (2012). The immune contexture in human tumours: impact on clinical outcome. *Nature Reviews Cancer*.

[21] Galon J., Pages F., Marincola F. M. (2012). Cancer classi cation using the Immunoscore: a worldwide task force. *Journal of Translational Medicine*.

[22] Lea D., Haland S., Hagland H. R., Soreide K. (2014). Accuracy of TNM staging in colorectal cancer: A review of current culprits, the modern role of morphology and stepping-stones for improvements in the molecular era. *Scandinavian Journal of Gastroenterology*.

[23] Mlecnik B., Tosolini M., Kirilovsky A. (2011). Histopathologic-based prognostic factors of colorectal cancers are associated with the state of the local immune reaction. *Journal of Clinical Oncology*.

[24] Anitei M.-G., Zeitoun G., Mlecnik B. (2014). Prognostic and predictive values of the immunoscore in patients with rectal cancer. *Clinical Cancer Research*.

[25] Galon J., Costes A., Sanchez-Cabo F. (2006). Type, density, and location of immune cells within human colorectal tumors predict clinical outcome. *Science*.

[26] Sato E., Olson S. H., Ahn J. (2005). Intraepithelial CD8^+^ tumor-infiltrating lymphocytes and a high CD8^+^/regulatory T cell ratio are associated with favorable prognosis in ovarian cancer. *Proceedings of the National Acadamy of Sciences of the United States of America*.

[27] Spranger S., Spaapen R. M., Zha Y. (2013). Up-regulation of PD-L1, IDO, and T(regs) in the melanoma tumor microenvironment is driven by CD8(+) T cells. *Science Translational Medicine*.

[28] de Guillebon E., Roussille P., Frouin E., Tougeron D. (2015). Anti program death-1/anti program death-ligand 1 in digestive cancers. *World Journal of Gastrointestinal Oncology*.

[29] Muro K., Chung H. C., Shankaran V. (2016). Pembrolizumab for patients with PD-L1-positive advanced gastric cancer (KEYNOTE-012): a multicentre, open-label, phase 1b trial. *The Lancet Oncology*.

[30] Diaz L. A., Le D. T., et al. (2015). PD-1 blockade in tumors with mismatch-repair deficiency. *The New England Journal of Medicine*.

[31] Taube J. M. (2014). Unleashing the immune system: PD-1 and PD-Ls in the pre-treatment tumor microenvironment and correlation with response to PD-1/PD-L1 blockade. *OncoImmunology*.

